# MetLab: An *In Silico* Experimental Design, Simulation and Analysis Tool for Viral Metagenomics Studies

**DOI:** 10.1371/journal.pone.0160334

**Published:** 2016-08-01

**Authors:** Martin Norling, Oskar E. Karlsson-Lindsjö, Hadrien Gourlé, Erik Bongcam-Rudloff, Juliette Hayer

**Affiliations:** 1 National Bioinformatics Infrastructure Sweden (NBIS), Uppsala University, Uppsala, Sweden; 2 SLU Global Bioinformatics Centre, Department of Animal Breeding and Genetics (HGEN), Swedish University of Agricultural Sciences (SLU), Uppsala, Sweden; 3 Department of Biomedical Sciences and Veterinary Public Health (BVF), Swedish University of Agricultural Sciences (SLU), Uppsala, Sweden; 4 The OIE Collaborating Centre for the Biotechnology-based Diagnosis of Infectious Diseases in Veterinary Medicine, Uppsala, Sweden; University of Arizona, UNITED STATES

## Abstract

Metagenomics, the sequence characterization of all genomes within a sample, is widely used as a virus discovery tool as well as a tool to study viral diversity of animals. Metagenomics can be considered to have three main steps; sample collection and preparation, sequencing and finally bioinformatics. Bioinformatic analysis of metagenomic datasets is in itself a complex process, involving few standardized methodologies, thereby hampering comparison of metagenomics studies between research groups. In this publication the new bioinformatics framework MetLab is presented, aimed at providing scientists with an integrated tool for experimental design and analysis of viral metagenomes. MetLab provides support in designing the metagenomics experiment by estimating the sequencing depth needed for the complete coverage of a species. This is achieved by applying a methodology to calculate the probability of coverage using an adaptation of Stevens’ theorem. It also provides scientists with several pipelines aimed at simplifying the analysis of viral metagenomes, including; quality control, assembly and taxonomic binning. We also implement a tool for simulating metagenomics datasets from several sequencing platforms. The overall aim is to provide virologists with an easy to use tool for designing, simulating and analyzing viral metagenomes. The results presented here include a benchmark towards other existing software, with emphasis on detection of viruses as well as speed of applications. This is packaged, as comprehensive software, readily available for Linux and OSX users at https://github.com/norling/metlab.

## Introduction

Metagenomics is the study of the combined genomic material of a sample and as such viral metagenomics concentrate on characterization of the viral fraction of the sample.[1,2]. By applying high throughput sequencing (HTS), with selective enrichment of the viral target and reduction of host genome, the virome of an organism or environment is explored in an unbiased way, without the need for culturing or viral isolation. The use of this methodology have increased enormously in the last decade, partially due to the increased availability of high throughput sequencing, but also due to development of better tools for analysis and interpretation of datasets [3–5]. For viral discovery the availability of the technology has dawned a new era, with several notable findings the last 10 years [6]. The technology is also in use for disease monitoring, investigations into complex multifactorial disease and for preparedness against new zoonotic agents [2,6–9].

Due to the complexity of metagenomic investigations, several caveats exist for designing experiments and analyzing their results. The three most common problems are related to the nature of the target. i), Viruses are small genomic entities within a world of giants; even the smaller bacterial genomes are considerably larger and risk masking out the viral genomes during analysis [10]. This is normally solved by either viral enrichment, e.g. DNA amplification or virus isolation, or by increasing the depth of sequencing [11]. ii), the diversity of viral genomics is incredibly complex, encompassing all known variants of genomic structure [12]. iii) The current knowledge of viral diversity, e.g. studies estimate that as much as 95% of the viral diversity as unknown, providing a huge range of unknown factors while performing analysis [10,2]. This leads to problems in estimating viral abundance in a sample as well as problems related to the availability of target sequences while assigning taxonomic identities to the sequence reads during analysis [5].

### Coverage theories and its application within metagenomics

In metagenomic experiments the coverage of each genomic entity within the sample is one of the few metrics available to estimate how good the dataset is [12]. With high enough sequencing depth, the identification of all genomes within the metagenome is feasible and as such the estimated coverage of genomic entities within a dataset is an important metrics for determining the validity of an experiment [13]. Coverage theories for metagenomics try to assess the needed depth of sequencing within an experiment [14]. This will enable researchers to evaluate the metagenomics dataset as an objective subsample of the metagenome e.g. as with the common estimate of sample size to reach coverage of a population [13–15]. This enables a researcher to estimate not only the needed amount of sequencing data, but indirectly also predict the lowest abundance genome that can likely be identified with a given sequencing technology [12]. For these calculations to be valid there must be good estimates of the genome size range within the sample, the abundance of the different species and the sequencing output [14,15].

### Bioinformatics analysis of viral metagenomes

#### Quality control

The sequence quality of HTS data is of great importance for the validity of the results within an metagenomics experiment, the introduction of low quality datasets will not only increase the complexity of the analysis, it will also risk producing false positives e.g. known viruses classified as new strains due to erroneous reads [16,17]. For viral metagenomics, sequence contamination is often introduced during the nucleotide amplification, during either the selective enrichment procedure or during the library preparation [16,17]. This data corruption combined with technology specific systematic errors, e.g. the Illumina GGC error, must be removed or accounted for during analysis [18,19]. Even though several tools exists for measuring sequence read quality, only one tool includes metrics specifically for metagenomics: PrinSeq [20–22].

#### Assembly

Genome assembly has historically been focused on single genomes [23]. As such the focus has been high sequencing depth, good mean coverage and removal of contaminating sequence reads e.g. alien sequences [20]. In metagenomics assemblies however, the focus of the dataset is multiple genomes, low mean coverage and plenty of contaminating sequence i.e. from host or species outside the focus of the study, like bacteria in viral metagenomics datasets [4]. The complexity of species diversity within the sample, as well as low coverage, introduces problems with chimeric contigs e.g. the synthetic combination of reads from two or more organisms genomes, which increases the complexity of the assembly as well as provides possible false positives in downstream applications [24–26]. Different approaches can be used to limit the complexity of the dataset, including mapping towards reference sequences to remove known species within the sample [27].

Almost all *de novo* assemblers build on one of three themes; i) the greedy algorithm e.g. CAP3 and TIGR, ii) the Overlap-Layout-Consensus e.g. Celera assembler, Mira and Newbler, and iii) strategies based on de Bruijn graphs e.g. SPAdes and Ray [28,29] [30–33]. For metagenomics datasets there is also a number of adaptions of existing software as well as some specialized methodologies available for *de novo* assembly [29,34,35]. It is estimated that over 90% of the microbial genomes are undiscovered, and in addition, the included genomes are unknown, making mapping assembly impossible. Thus *de novo assembly* is the standard approach to metagenomics datasets [36].

#### Taxonomic binning

The characterization of the taxonomic diversity of microbial communities is one of the primary objectives in a metagenomic study [10]. Phylogenetic classification of metagenomic reads, referred to as binning, is a problem closely related to assembly [4,5,37].

Several binning methods have been developed, and can be categorized as two types: taxonomy-dependent or taxonomy-independent [38]. Taxonomy-dependent methods aim to classify sequences into known taxonomic groups, by following supervised learning procedures, while taxonomy-independent methods, aim to bin the reads based on mutual similarity, without database comparison. Taxonomy independent methods are thus closely related to unsupervised machine learning procedures [37].

Taxonomy-dependent methods can be divided into three subclasses: alignment-based methods, composition-based methods, and hybrid methods, using both alignment and composition for the binning [37,38]. Alignment based methods commonly rely on BLAST, followed by applications of the Lowest Common Ancestor Algorithm to classify the reads in taxonomic groups [39,40]. A limitation of Blast-based approaches is the computing cost. To combat this limitation, several methods have been developed to speed up the process, introducing tools such as Kraken, Diamond [41] and GPU-BLAST [42–44].

Composition-based methods instead use compositional properties like GC-content, oligonucleotide usage, or codon-usage patterns to classify reads, based on models or sequence motifs from a reference database [45]. Hybrid methods use a combination of alignment and composition based methods. For example, PhymmBL combines the results of BLAST with scores produced from Interpolated Markov Models, aiming to achieve higher accuracy than BLAST alone [46].

#### Viruses vs bacteria

To our knowledge, the only standalone approach developed to classify viral sequences is ProViDE [47]. Most binning approaches are based or trained on bacterial marker genes, thus are mainly useful for bacterial sequences. Indeed, the study of viral diversity is hampered by the lack of universally conserved genes across all viral species, such as the 16s ribosomal RNA gene in prokaryotes or the Internal transcribed spacer in fungal eukaryotes [2,48].

As previously mentioned, HTS based metagenomics approaches have been used to great success during the last decade for viral discovery [7]. The methods used are however limited by the stringency of taxonomy based methods, and 60–99% of the sequences generated in different viral metagenomic studies are not homologous to known viruses [6], providing a challenge for identification and characterization of new viruses.

#### Aim

This study aims at producing a bioinformatics framework for design and analysis of viral metagenomics experiments, MetLab. This is done by providing an implementation of the algorithms proposed by Wendl *et al*. [14] for estimating needed coverage, simulating viral metagenomes, as well as providing analysis pipelines for i) preprocessing of datasets, ii) elimination of host material and iii) quick taxonomic classification. This is packaged, as a comprehensive piece of software, readily available for Linux and OS X users and with a graphical user interface from https://github.com/norling/metlab.

## Material and Methods

MetLab is written as a two-part application, the computational framework and the graphical user interface (GUI). The framework is composed of three main modules. Each module can be used via the GUI (a python tkinter interface (http://tkinter.unpythonic.net/wiki/)), and both the Metamaker and the Experimental Design modules can be used as standalone command-line applications, which provide an easy-to-use alternative.

The application is written in Python 2.7 (www.python.org/), giving platform independence, and is released under the GPLv3 license, allowing any developer to extend or incorporate the classes into future systems. The included modules so far are: a viral metagenomic dataset simulator, a coverage probability module for experimental design, and an adaptable analysis pipeline module.

### Metamaker module: viral datasets simulation

The Metamaker module has two functions; it can read a set of sequencing data, creating a statistical profile, and secondly, simulate datasets from such a profile. The profile includes read length, read length variation, number of reads, and per-base error probabilities. The profile is designed to be simplistic but create a reasonable approximation of real sequencing data. The user can choose from seven sequencing technologies profiles that are currently available IonTorrent, IontProton, Illumina MiSeq, Illumina HiSeq, Illumina NextSeq, Pacific Biosystems and Oxford Nanopore. To generate a dataset—the user inputs the number of species to include and the distribution of their abundance (uniform or exponential), and the module downloads random viral genomes from NCBI, generating two output files. One is a Sanger Fastq file with read statistics corresponding to the profile, and the other one is a key file, a list of comma-separated values (csv) describing the dataset contents.

The script introduces errors randomly according to the quality values, marking erroneous nucleotides in the fastq sequence using lowercase letters. The script makes heavy use of BioPython (biopython.org) for communicating with NCBI, downloading and parsing sequence data, and uses the numpy library (www.numpy.org) for efficient numerical calculations.

### Experimental design module: implementation of Stevens Theorem

There are multiple proposed ideas for estimating the sequencing needs of a metagenomic project. One of the more advanced algorithms for calculating this need was published by Wendl *et al*., [14], which is an adaptation of Stevens’ theorem. A metagenomic assembly starts with a number of reads, **R**, of (mean) length **l** from a metagenomic community. The probability of assembling a certain member of the metagenomic community with an abundance of **α** and a genome size of **L** can then be calculated. The probability of a position in the target genome being covered by a read from the same genome can be written as **φ** = (**l**/**L**). Together with the Steven’s series limiter *η* = min (**R**, int(1/ **φ**)) this is used to calculate the probability of an ideal assembly with *k* gaps, according to the algorithm proposed by Wendl, *et al*. The module includes implementations of theorem 1 (gap consensus), Eq 1, and it’s first corollary for calculating the probability of complete coverage (Eq 2).

$$P\left(B=k\right)=\left(\begin{array}{c} R\\ k \end{array}\right)\overset{\eta }{\underset{\beta =k}{\sum }}\left(\begin{array}{c} R-k\\ \beta -k \end{array}\right){\left(-1\right)}^{\beta -k}\alpha^{\beta }{\left(1-\beta \varphi \right)}^{\beta -1}{\left(1-\beta \varphi \alpha \right)}^{R-\beta }$$ (1)

Stevens’ theorem for metagenomic gap consensus probability [14], B describes the number of sequence gaps in a theoretical ideal assembly of k gaps, where R is the number of sequence reads, φ is the probability of a position being covered, α is the species abundance in the community and η is the smaller of R and int(1/φ).

$$P\left(B=0\right)=\;\overset{\eta }{\underset{\beta =0}{\sum }}\left(\begin{array}{c} R\\ \beta \end{array}\right){\left(-\alpha \right)}^{\beta }{\left(1-\beta \varphi \right)}^{\beta -1}{\left(1-\beta \varphi \alpha \right)}^{R-\beta }$$ (2)

Stevens’ theorem for metagenomic full coverage probability [14], where R is the number of sequence reads, φ is the probability of a position being covered, α is the species abundance in the community and η is the smaller of R and int(1/φ).

These are computationally hard problems, which regular precision programs do not handle well. In response to this, a dedicated python module was implemented as a C extension using the GNU MPFR Library [49], a multiple-precision floating-point library allowing arbitrary numerical precision calculations, as well as mpmath (http://mpmath.org/), a python library giving a slower, but easier to install, solution.

#### Analysis pipeline module

The metagenomic analysis pipeline, based on a set of programs suited for metagenomic analysis, is modular and as such flexible depending on the users need for analysis, e.g. omitting assembly and or host filtering. The pipeline starts with data pre-processing with Prinseq-Lite [50]. Trimming and filtering options are set to default values (extrapolated from a normal need), but the user can easily modify them. The next steps is host genome mapping with Bowtie2 [51], designed for metagenomic analysis from animal samples. Reads that do not map to the host genome are extracted using SAMTOOLS [52], *de novo* assembly is performed on the unmapped reads with SPAdes [28], which is optional and must be enabled by the user. The unmapped reads, and possibly contigs, are then taxonomically classified.

#### Selection of methods for taxonomic classification

At the start of the developing process, the door was open to several classification methods, the goal being to find the best compromise between ease of use, speed and accuracy. Eight datasets were simulated with the Metamaker module using Ion Torrent and Ion Proton profiles, with different read lengths and species distributions. All the reads with a mean Phred quality score < 20 were discarded using PrinSeq. These simulated datasets were used to benchmark several metagenomics taxonomic binning tools: Kraken [42], Blastn + LCA from the Fragment Classification Package (FCP) [53], Diamond (blastx command) [41], Blastx and MEGAN 5 [40], RAIphy [54], ProViDE [47] and Naïve Bayes Classifier (NBC) [55].

As most binning methods are unable to work on short reads, all datasets were assembled *de novo* using Ray. Chimeric contigs, which corresponded to an average of 7% of the contigs across the 8 datasets, were discarded and the contigs originating from only one species were classified using the previously cited binning methods.

All the classifiers were used with default parameters, and the following databases: nr (NCBI) for Diamond and Blastx, a combination of RefSeq (Archea + Bacteria) and the phages and viral divisions of GenBank for Kraken and RAIphy, and the NCBI Archaeal, Bacterial and Viral genomes for FCP and NBC.

In the case of Kraken, the database was used i) as-is (superDB) ii) using the—max-db-size parameter with a value of 4G (minisuperDB or shrunk database).

In the implemented pipeline, after binning with the method of choice, Krona Tools is used to generate a HTML report summarizing the taxonomic classification as an interactive pie chart [56].

The reads that remained unclassified after primary binning classification are retrieved for a classification at protein level to be able to detect more divergent homologies. Gene prediction is attempted using FragGeneScan [57], which provides predicted protein sequences as output. These predicted protein sequences are then scanned for a set of viral Hidden Markov Models (HMM) profiles called vFams, published by Skewes-Cox *et al*. [58], using HMMER3 [59]. Results are summarized in a report, listing sequences with a significant match with a vFam, the corresponding vFam, and the viral families of sequences used to build the vFam profile. These predictions can also be visualized in a Krona chart produced by the module. The system also provides the user with output files that are tab-separated and can be imported in Excel or R for further analysis.

### Testing on published datasets

In order to test our analysis pipeline, two other sets of data were used as a comparison in this study; one being a simulated dataset published in the Clinical Pathoscope article [60] and the other being a real dataset classified using a BLAST-LCA approach.

The first dataset used is a simulated dataset from the Clinical Pathoscope project, containing human (90%), bacterial (9%) and viral reads (1%). After quality control, the good quality reads were mapped toward the human genome following the MetLab standard procedure before to be classified using Kraken.

The second data-set used was the initial dataset used for the publication “Metagenomic Detection of Viral Pathogens in Spanish Honeybees: Co-Infection by Aphid Lethal Paralysis, Israel Acute Paralysis and Lake Sinai Viruses” [61]. In this publication a single Roche 454 Life Science run on one eight of a Pico titer Plate. For a more detailed study see the original publication. Dr. Fredrik Granberg, SLU, Sweden graciously provided this dataset. The aim was a direct comparison of the results gained from that study with results gained from the approach presented in this article.

## Results and Discussion

The developed software, Metlab, consists of several modules implemented within a framework to simplify design, simulation and analysis of metagenomics datasets, with emphasis on detecting previously known and putatively novel viruses. The read simulation module, Metamaker, is implemented to provide a preliminary dataset for scientists to estimate the complexity and validity of the different analytical pipelines. The second module provides confidence values for detecting all viral genomes within a sample, based on the generalization of Steven’s Theorem. This enables the user to make an informed decision when designing the sequencing part of the experiment and as such avoid the possibility of under/over-sequencing the sample. The third module is dedicated to the analysis of the dataset; incorporating quality control, host filtering, assembly and taxonomic classification.

### Metamaker module: viral datasets simulation

The Metamaker module reads sequencing data, generates profiles from the data and simulates read sequences based on the profile and NCBI viral sequences. It can generate datasets of Ion Torrent, Ion Proton, Illumina MiSeq, Illumina HiSeq, NextSeq as well as Pacific Biosystems and Oxford Nanopore profiles.

The module produces viral datasets with realistic error profiling and known taxonomic content, enabling testing and validation of assembly and taxonomic binning methods.

### Experimental design module: implementation of Stevens Theorem

The model proposed by Wendl *et al*. was previously not implemented. A novel implementation was developed, relying on the GNU MPFR Library, written in C, dedicated as a Python module. This allowed the implementation of the proposed model as well as maintaining ease of installation and providing users with a confidence value based estimation of the needed sequencing depth for a metagenomics experiment.

The implementation estimates the needed sequencing depth based on the Metamaker profiles produced while simulating datasets. Given the lowest species abundance and its genome size, the module calculates the probability of covering all included genomes (such as at least one contig is produced from each genome) given a theoretical optimal assembly. If a single run is not sufficient to reach that probability the module goes into iterative state, consecutively adding simulated runs until coverage probability is reach or a maximum of 10 runs are simulated.

The experimental design probability calculations can either be used from the command-line, or from the graphical user interface.

### Analysis pipeline module

#### Selection of a binning method

Eight datasets simulated using Metamaker were used for comparing taxonomic methods and selecting the one method to be integrated into the analysis module. Basic read and assembly statistics for the 8 simulated datasets are shown in Table 1. A direct comparison of the six methods running time and system resources needed is shown in Table 2. Only Kraken and RAIphy ran in less than one hour, while Diamond ran in 3.3 hours on 4 cpus, and Blastx for over five days on 8 cpus. However, Kraken used 78G of RAM with the superDB, more than 25 times than RAIphy. The use of the shrunk database (superminiDB) greatly reduced the amount of RAM needed by Kraken to 4.5G.

**Table 1:** Statistics of the simulated reads: quality filtering and de novo assembly.

	NGS profile	IonProton		IonTorrent200		IonTorrent400		IonTorrent	
	Species Distribution (200 viruses)	*Exponential*	*Uniform*	*Exponential*	*Uniform*	*Exponential*	*Uniform*	*Exponential*	*Uniform*
Prinseq quality filtering	**Number of input sequences**	15,399,727	14,553,370	591,020	610,006	411,304	462,169	2,521,607	2,623,306
	**Input mean length (nt)**	144.64	144.65	244.1	243.89	325.19	325.52	198.88	198.71
	**Good sequences (%)**	84.64%	85.73%	88.49%	88.45%	83.88%	83.86%	85.87%	85.89%
	**Good sequences mean length (nt)**	153.68	152.92	237.54	237.48	336.88	336.97	215.14	215.14
Ray *de novo* assembly	**Number of contigs**	2,455	3,521	1,953	7,533	2,659	8,889	1,111	3,075
	**Total length (nt)**	2,939,578	6,220,833	2,361,974	6,777,218	2,269,692	6,662,688	1,655,583	6,146,569
	**Average length (nt)**	1,197	1,766	1,209	899	853	749	1,490	1,998
	**N50 (nt)**	24,608	25,523	11,761	2,077	3,566	1,350	25,150	12,567
	**Largest contig (nt)**	171,369	167,708	93,770	59,708	159,613	49,652	137,229	93,761
	**Used reads (%)**	89.93	95.93	98.2	94.37	97.98	91.51	31.19	91.35

**Table 2:** Comparison of time and computing resources used by the compared binning methods.

	Kraken (superDB)	Kraken (minisuperDB)	RAIphy	FCP Blastn+LCA	Diamond (Megan)	Blastx (Megan & ProViDE)	NBC*
**Mean running time**	33 mins	< 1 min	30 mins	75 mins	3.3 hrs	> 5 days	NA
**Memory required**	78G	4.2G	3G	2.5G	9.5G	10G	NA
**CPUs used**	1	1	1	8	4	8	NA

*These data are not available for NBC as it was run online.

**Fig 1:**
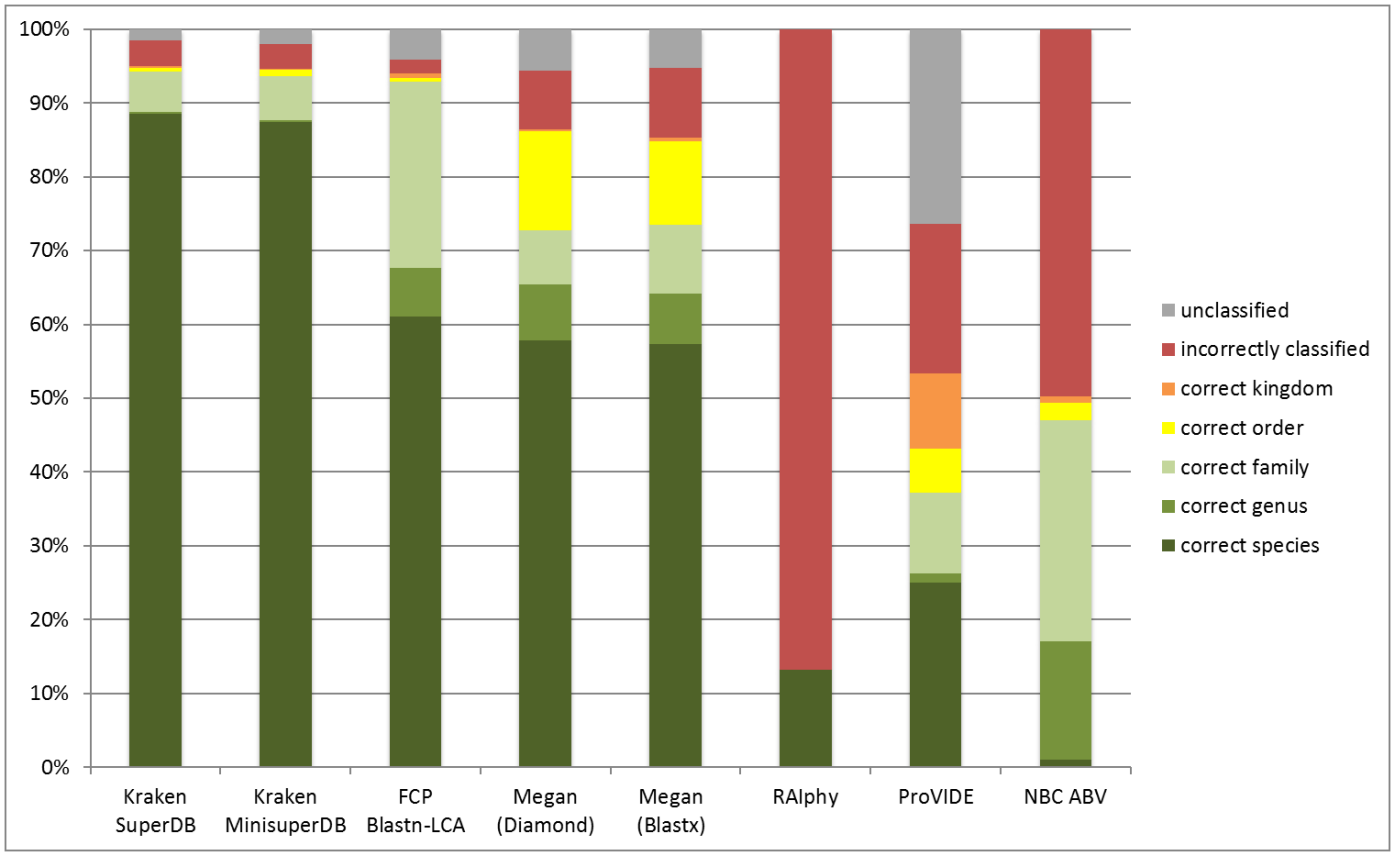
Comparison of the binning methods. The represented percentages are an average of validations obtained with the 8 simulated datasets.

For each binning method, the assigned taxonomy of each contig was compared to its actual taxonomy. The results are summarized in Fig 1.

The analysis shows that after validation, Kraken outperformed all the others methods, classifying 88.58% of the contigs at the correct species. When using the shrunk database, Kraken classified correctly 87.36% of the contigs while using 17 times less RAM. The Blast-based methods also performed well, with about 60% of the sequences classified at the correct species, with Blastn+LCA (FCP) classifying 93% of the contigs at the correct family. Megan5 used either with Diamond or Blastx achieved a similar level of accuracy at the species level but classified less viruses at the family level and had a higher level of false positives (7.90% for Diamond, 9.45% for Blastx). ProViDE, also based on Blastx, showed less accurate predictions than Megan, with less than 40% of the contigs classified at the correct family.

It has to be noted that the NBC tool and RAIphy always give a prediction, hence the percentage of unclassified sequences by these methods is 0. Notably, RAIphy always provides a prediction at the species level. Thereby the predicted species was wrong 86% of the time.

These results show that not all kinds of binning methods are well adapted to the classification of viral sequences and that the most efficient methods are the alignment-based methods. Methods dedicated to virus detection and using only viral dataset can be biased and then over-assign some sequences, producing a high amount of false positives in the results. Moreover, using a tool than can detect viruses as well as archaea and bacteria has its use even if the purpose of the analysis is to detect the viruses, because it enables to detect the possible bacterial contaminants. Of the two best performing alignment-based methods, providing the largest amount of correctly classified sequences, Kraken outperforms the secondary method, Blastn-LCA. This is true for both running time as well as accuracy. Kraken has proven to be both efficient and effective in performing classifications, as well as having the benefit of being able to quickly analyze a huge amount of sequences, making it possible to run without the assembly step.

Kraken being efficient in classifying short sequences, it was also run on the reads using the shrunk database. 86.03% were classified at the correct species, a level of accuracy similar to the analysis carried on the contigs, and using the same amount of computing resources.

Seeing that Kraken was i) the most accurate, ii) the fastest and iii) able to run on a consumer grade laptop using the shrunk database, it was seen as the best choice for primary taxonomic binning method and integrated into MetLab. Indeed, Kraken is included as a classifier, together with two separate databases; the expanded viral database, including all data from the VRL and PHG divisions of GenBank as well as RefSeq Archaea and Bacteria, and the shrunk version of the same, suitable for running classifications on consumer grade computers. As the other classifiers were deemed unsuitable for this application they were excluded from the software.

#### Detection of highly divergent viruses

A secondary method using HMMER on the vFam database was implemented to predict the reads of viral origin where Kraken could not get a clear match. Working at the protein level for detecting sequences of viral origin is a logical step as Kraken requires exact matches and is highly dependent on the database used for classification. As such, using a secondary method on the unclassified reads enables the users a higher sensitivity for detection of viral families and detection of previously uncharacterized viruses.

However, that secondary method could not be used on the simulated datasets. Indeed, almost no reads remained unclassified after running Kraken. This case will not arise when using real datasets, which contain more sequences from unknown viruses. The method will prove itself useful on the tests realized on existing real datasets.

### Testing on published datasets

#### Testing on the Clinical Pathoscope dataset

After quality filtering and removal of the human sequences, a total number of 970602 reads were analyzed with Kraken. About 74% of those reads were classified as bacteria, 17% of the reads remained unclassified and 9% were classified as viruses. Within the 74%, the reads were correctly classified at least to the genus level, with 41% of reads classified to the genus *Streptococcus*, 39% to the *Haemophilus* genus and 19% to *Moraxella*, which corresponded to the actual proportions of these genera in the dataset. After validation of the classification of viral reads, 4.80% of the viral reads were unclassified and among the 95.2% of classified viral reads 93.35% were predicted at the correct family, with 87.95% at the correct species, and 1.85% were wrongly predicted

#### Testing on the “Spanish Honeybees” dataset

Summarized in Table 3 are the comparison of viral read detection between the Blastn-LCA approach and the approach used within MetLab (Kraken and vFam) for the dataset from Granberg *et al*. The three main viruses found are the same with both methods. Focusing on the ssRNA viruses, a direct comparison provides some valid questions. In the original analysis several reads were classified as Turnip Yellow Mosaic Virus (TYMV), a virus belonging to the Tymovirus, but this virus could not be identified using Kraken. However, Granberg *et al*. state in the publication that *for TYMV*, *one contig of 225 bp was generated and it shared 91% nucleotide sequence similarity with its most similar reference genome (GenBank X07441)*, *but only over a stretch of 56 bp in the middle*. *Since the ends did not show any resemblance with the reference*, *this could either indicate a new type of TYMV-like virus or an incorrectly assembled contig*. 206 reads similar to two vFams containing sequences only from Tymoviridae were found by MetLab. This additional information brought by the analysis at the protein-level indicates that a virus distantly related to the TYMV could be present in the dataset. Moreover, MetLab, with its combined prediction method, enabled the detection of sequences from Baculoviridae and Phycodnaviridae, as well as other viral families.

**Table 3:** Viral sequences detected in the Spanish Honeybees dataset. Comparison of the number of reads classified as viruses by Granberg et al. (Blastn-LCA method) and the number of reads classified as viruses by MetLab with Kraken and vFam methods.

	Granberg *et al*.	MetLab results		
Taxon	Blastn-LCA	Kraken	vFam	MetLab total
Secoviridae	1968 (TuRSV)	936 (TuRSV)	279	1215
Dicistriviridae	1048 (IAPV)	583 (IAPV)	0	583
	664 (ALPV)	878 (ALPV)	0	878
Tymoviridae	563 (TYMV)	0	206	206
Caudovirales (Phages)	30	22	0	22
Retroviridae	16	68	0	68
Lake Sinai Virus	14	38	0	38
Baculoviridae	0	11	535	546
Phycodnaviridae	0	8	193	201
Others	7	769	613	1382
*Total viruses reads*	*4310*	*3313*	*1826*	*5139*

TuRSV: Turnip Ringspot Virus, IAPV: Israel Acute Paralysis Virus, ALPV: Aphid Lethal Paralysis Virus, TYMV: Turnip Yellow Mosaic Virus.

Concluding the results we can see that even though there are some minor drawbacks with the use of Kraken as a classification tool (see [42] as well as previous discussion) the main goal of rapid classification is achieved using this approach. Given the need of simplification of databases a rapid taxonomy dependent, highly specific classification tool will shorten analysis by several hours if not days. With the ultimate goal of providing a tool that is easy to use the format of Kraken results is likable, and a graphical representation in Krona [62] available within MetLab makes it even more user friendly.

**Fig 2:**
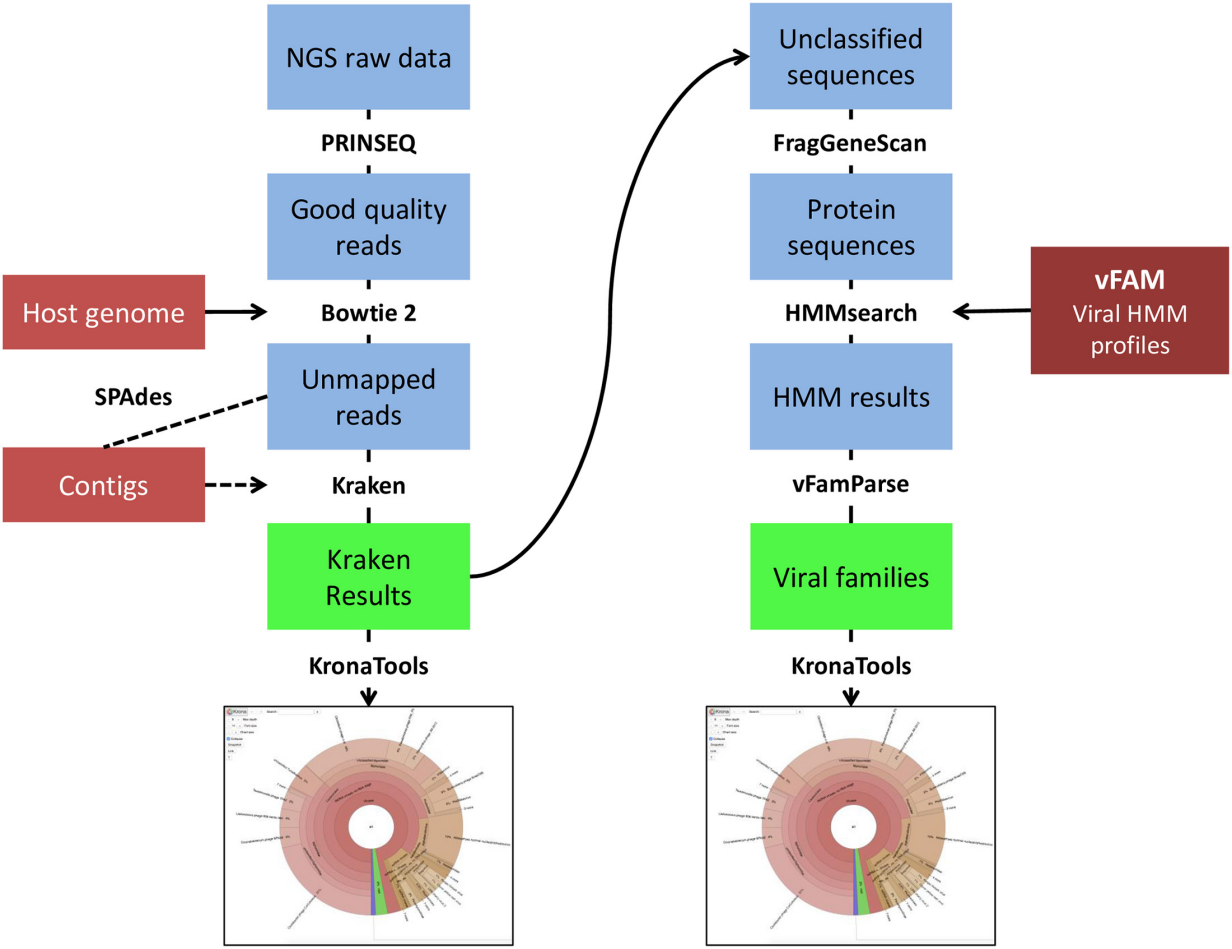
Main workflow of the MetLab analysis pipelines.

The addition of a protein level classification on all the viral reads left unclassified by Kraken using FragGeneScan and HMMER3 with the vFam database adds valuable information about viral families without adding much time to the analysis. The complete analysis workflow is presented on Fig 2.

### MetLab Graphical User Interface

**Fig 3:**
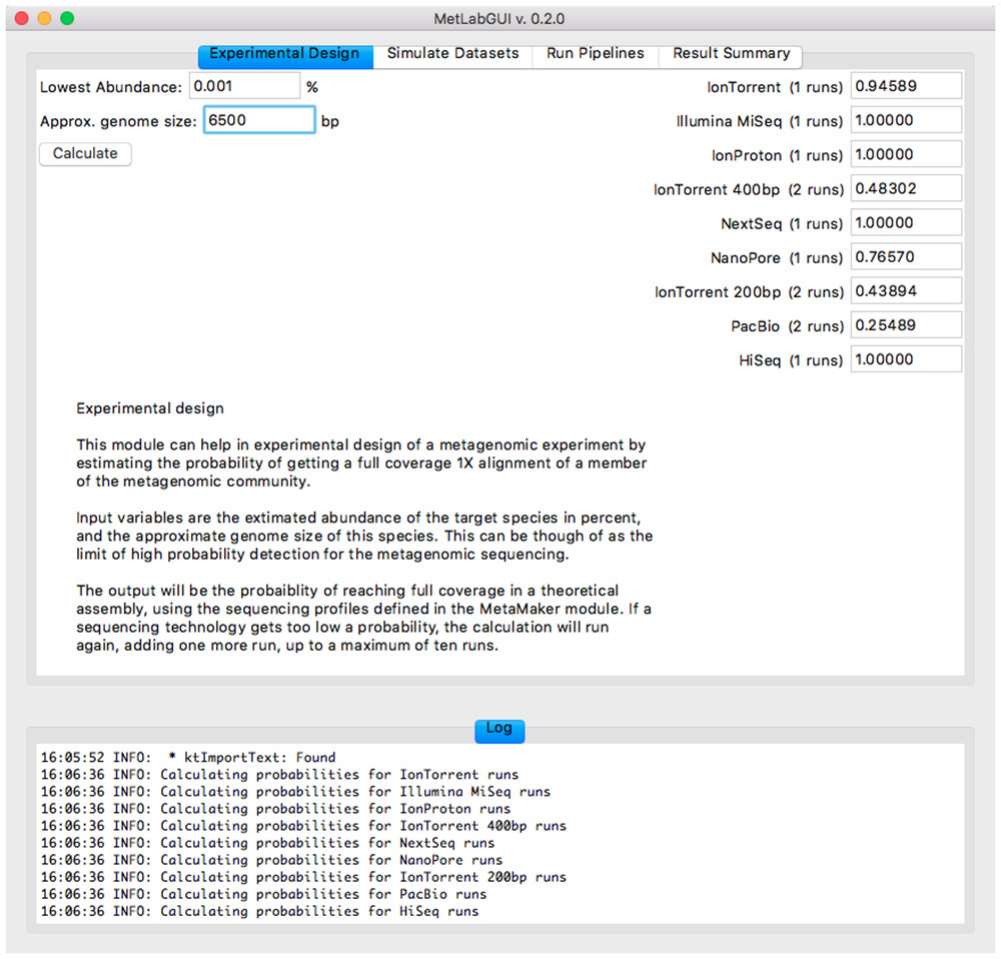
MetLab GUI: the experimental design module.

The experimental design module of the graphical user interface (GUI) is presented on Fig 3. The three modules of MetLab are displayed selecting tabs, accessible at a glance. All the parameters and options present at the command-line are accessible through the GUI for each separate module.

The standard analysis pipeline is implemented without assembly but the user can easily switch to a pipeline with an assembly step. The standard pipeline enables rapid classification of viral reads after quality filtering and host removal by mapping, by taxonomic binning using Kraken as well as prediction of sequences of viral origin on unclassified reads by use of vFam/HMM. Results are presented to the user both as Krona charts and as tab-separated files that can be imported in R for further analysis. By providing both a visual representation and a table the user is enabled to both have a quick overview of the results as well as an in-depth source of information for further investigations.

## Conclusion

MetLab thus provides a wide field of usage, before, during and after the metagenomics experiment. It gives the opportunity to design the experiment, providing calculations for the coverage needed, enabling the user to carefully prepare his experiment depending on the sequencing technology used. MetLab also provides the Metamaker module, allowing the user to simulate viral metagenomics datasets with seven different sequencing technology profiles. Metamaker is useful for testing, validating and selecting external analysis tools that could be applied on the data. After the sequencing MetLab offers a panel of pipelines dedicated to the analysis of metagenomes. These pipelines go from pre-processing step to taxonomic classification. Several binning methods were tested throughout the course of the development and Kraken was chosen as a primary binning method with additional support gained from a taxonomic prediction at the protein level using HMMER and vFam, a database of viral profiles. Improvements may be achieved in order to detect a wider range of species, by working on the database used for taxonomic binning. Options to allow the user to extract reads of interest will be added in the near future. MetLab has already successfully been used internally for several studies, including “The intestinal eukaryotic virome in healthy and diarrhoeic neonatal piglets” [63].
